# Resection of metachronous pancreatic cancer 4 years after pancreaticoduodenectomy for stage III pancreatic adenocarcinoma

**DOI:** 10.1186/s12957-015-0712-5

**Published:** 2015-09-30

**Authors:** John B. Hamner, Michael White, Carly Crowder, Gagandeep Singh

**Affiliations:** Department of Surgery, City of Hope Comprehensive Cancer Center, Duarte, CA USA; Tulane University School of Medicine, New Orleans, LA USA

## Abstract

Pancreatic adenocarcinoma frequently recurs in patients even after resection with curative intent. The majority of these are early recurrences and are associated with metastatic disease, thus not amenable to repeat resection. Here we report a patient who underwent completion pancreatectomy for a metachronous pancreatic adenocarcinoma. This patient initially presented with painless jaundice and computed tomography (CT) revealed a mass in the head of the pancreas. Brushings obtained at endoscopic retrograde cholangiopancreatography (ERCP) were positive for adenocarcinoma. This patient then underwent a Whipple procedure and final pathology demonstrated stage III pancreatic ductal adenocarcinoma. Adjuvant therapy included gemcitabine and erlotinib. This patient was followed with physical examinations and serial laboratory and imaging studies. There was no evidence of disease for four years at which time and sharp elevation in CA-19-9 was found. Subsequent imaging revealed a mass in the remnant pancreas. Curative intent completion pancreatectomy was then performed which confirmed the presence of pancreatic adenocarcinoma. This was followed by adjuvant Gemcitabine based chemotherapy and chemoradiation. One year later the patient is alive with no evidence of disease. Thus, in highly selected patients with recurrent or metachronous pancreatic cancer, repeat pancreatectomy can be considered, but the course of treatment should be considered in a multidisciplinary setting.

## Background

In 2014, it was estimated that over 46,000 people would be diagnosed with pancreatic adenocarcinoma with greater than 39,000 patients dying from the disease [1] making it one of the more lethal malignancies encountered. Of the subset of patients who are resectable and undergo curative intend surgery, the majority will ultimately develop recurrent disease with approximately 80 % of recurrences occurring within the first 2 years [2, 3] with 5-year survival rates ranging from 5 to 40 % [4–6]. These recurrences may be isolated to the pancreatic bed or present as distant metastatic disease. Given the overall poor prognosis of this disease, there remains controversy regarding the role of surgery in recurrent disease. While most would agree that in patients with early recurrence, the role of surgery is limited at best, the treatment of late recurrences or metachronous tumors in the pancreatic remnant is less clear. Because the late development of a metachronous tumor, particularly one isolated to the pancreatic remnant is a relatively rare event, the role of surgery may be more easily justified. Here, a case is presented of a patient who developed a metachronous adenocarcinoma of the pancreas 4 years after the initial diagnosis of stage III pancreatic cancer, treated with upfront curative intent completion pancreatectomy followed by adjuvant chemotherapy and chemoradiation.

## Case presentation

**Fig. 1:**
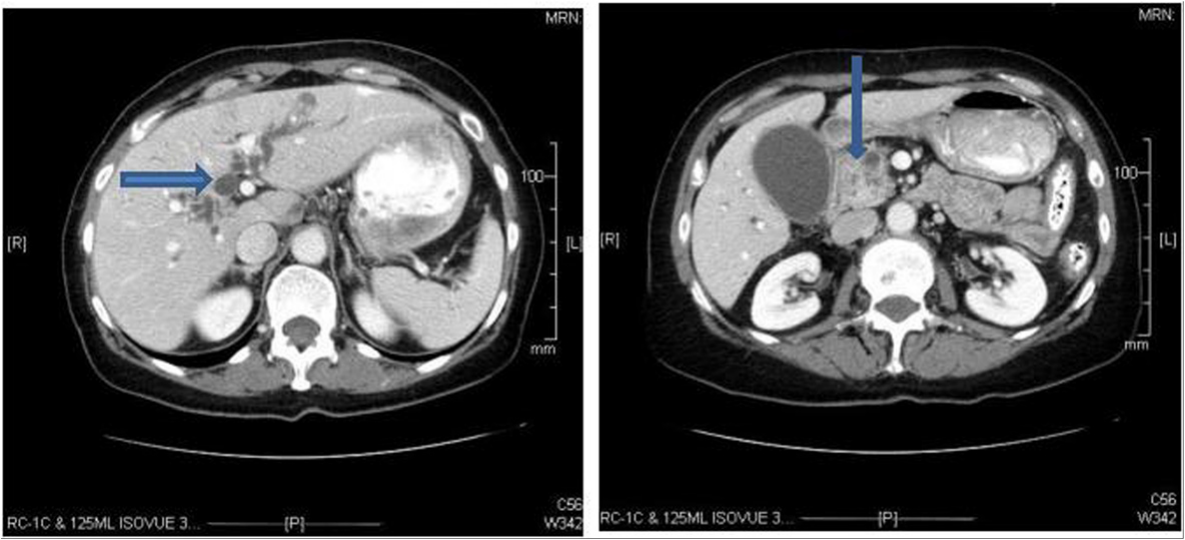
Representative cuts of the patient’s initial CT scan demonstrating intrahepatic biliary ductal dilatation (**a**) and a heterogeneous mass within the head of the pancreas (**b**)

In February 2010, a then 69-year-old female presented to her family physician with weight loss, early satiety and jaundice without abdominal pain, nausea, or vomiting. She was otherwise in excellent medical condition with her only significant past medical or surgical history being cervical carcinoma in situ treated with hysterectomy at age 29. She had no history of smoking and used alcohol only on rare social occasions. In addition, there was no family history of any malignant disease. Her initial workup included laboratory investigations that were significant for hyperbilirubinemia of 8.7 mcg/dL. This was followed by computed tomography (CT) of the abdomen and pelvis that revealed significant intra- and extrahepatic ductal dilatation (Fig. 1a) and a 2.69 × 2.64 cm mass in the head of the pancreas (Fig. 1b). There was no evidence of peripancreatic, periportal, mesenteric, or celiac axis adenopathy, nor was there evidence of distant metastatic disease. In addition, there was no abutment on the mass to the portal vein or the superior mesenteric artery and vein. Based on these initial CT findings, this was a resectable pancreatic head mass.

Her workup continued with endoscopic ultrasound (EUS) and endoscopic retrograde cholangiopancreatography (ERCP). These studies confirmed the presence of a mass within the head of the pancreas and demonstrated a 2-cm distal common bile duct stricture. Brushings of this area confirmed the presence of adenocarcinoma.

The patient was then referred for a surgical oncology evaluation. Repeat CT of the chest, abdomen, and pelvis was consistent with her original scan and showed no evidence of metastatic disease. Her CA 19-9 was checked and found to be elevated at 441 U/mL. Her case was discussed in a multidisciplinary tumor board, and because she appeared to be resectable at presentation, surgery rather than neoadjuvant therapy was offered.

In March of 2010, she was taken to the operating room where diagnostic laparoscopy revealed no evidence of metastasis and a classic pancreaticoduodenectomy was performed. Surgical pathology from the specimen showed a moderately differentiated infiltrating ductal adenocarcinoma of the pancreatic head. The tumor measured 4.2 × 3.0 × 2.6 cm and invaded into the peripancreatic soft tissue, though all margins were negative with the closest margin being 0.3 cm. Angiolymphatic invasion was identified. Two of 16 lymph nodes were positive for metastatic disease. Per the American Joint Committee on Cancer (AJCC) staging guidelines [7], this was a T3N1b, stage III pancreatic cancer. There was no evidence that this tumor was arising from a premalignant lesion such as an intraductal papillary mucinous neoplasm (IPMN). In addition, there was no mention of pancreatic intraepithelial neoplasia (PanIN) within the pancreas or at the margin in the original pathology report.

**Fig. 2:**
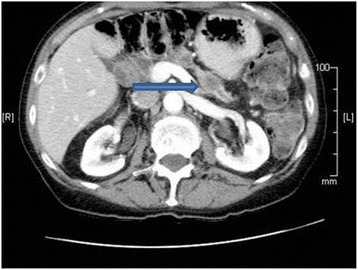
Representative cut of the patient’s CT scan in March of 2013, 4 years after the initial surgery. This demonstrates a mass within the pancreatic remnant suspicious for carcinoma

**Fig. 3:**
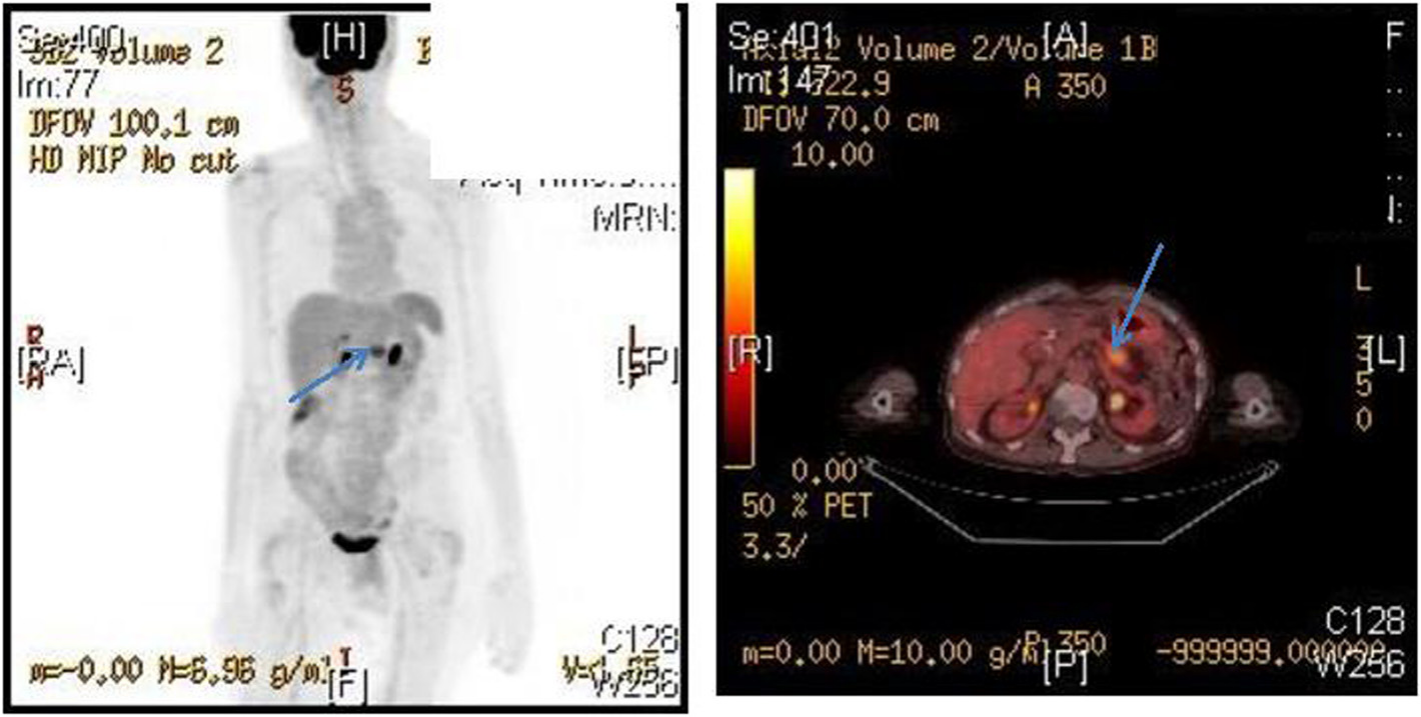
PET (**a**) and PET-CT (**b**) images demonstrating the mass within the pancreatic remnant with no evidence of metastatic disease

Postoperatively, she received adjuvant therapy with 3 cycles of gemcitabine monotherapy followed by an additional 3 cycles of gemcitabine with erlotinib. She underwent routine follow-up with physical examination, imaging, and laboratory studies on a semiannual basis. From late 2010 after completing adjuvant therapy until March 2014, she showed no evidence of disease with multiple negative imaging studies and CA19-9 levels ranging from 12.1 to 44.5 U/mL. In March 2014, however, her CA 19-9 increased to 161.4 U/mL. A CT done at this time (Fig. 2) showed a 2.1 × 2 cm mass in the body of the pancreas suspicious for recurrent or metachronous tumor. By CT, there was no evidence of disease outside of the pancreas. The CT was followed by PET-CT (Fig. 3) that demonstrated this PET avid lesion in the pancreas with a maximum SUV of 5.9 g/mL with no evidence of metastatic disease, again suspicious for pancreatic cancer. The patient’s case was presented at a multidisciplinary tumor conference with discussions on how to proceed with treatment, with the main questions focused on neoadjuvant therapy versus resection followed by adjuvant therapy if the mass is proven to be pancreatic carcinoma. Because of the long disease-free interval and the fact that the mass appeared resectable, the tumor board’s recommendation was to proceed with surgery first.

**Fig. 4:**
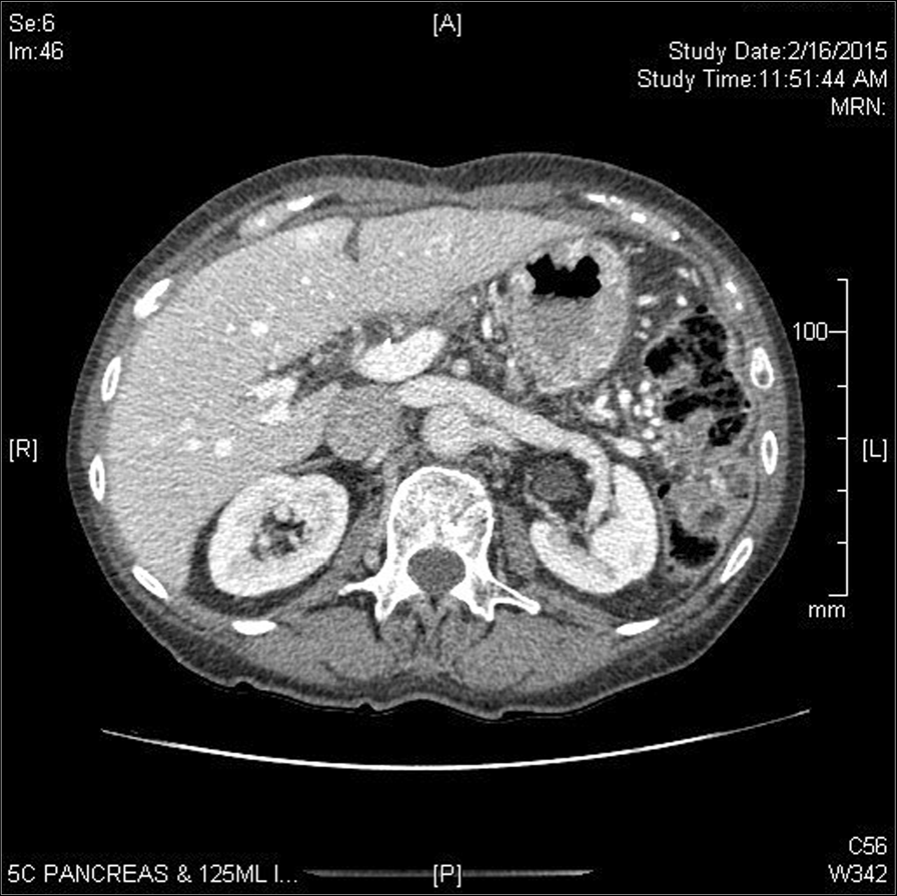
Follow-up CT 11 months after completion pancreatectomy. At this point, the patient has completed adjuvant chemotherapy and chemoradiation and there is no evidence of disease

In June 2014, the patient underwent a diagnostic laparoscopy that did not reveal evidence of disseminated disease, followed immediately by exploratory laparotomy with completion total pancreatectomy with splenectomy. This procedure lasted just over 3 h and was completed with minimal blood loss.

Surgical pathology from this operation demonstrated moderately differentiated ductal adenocarcinoma of the pancreas measuring 3.2 × 2.3 × 2.1 cm. All margins were negative. All lymph nodes evaluated (*n* = 7) were negative for metastatic disease. Based on AJCC staging criteria, this metachronous tumor was T2N0 or stage IIB. The patient’s hospital course was uncomplicated, and she was discharged home on postoperative day 7.

Following surgery, she received adjuvant therapy consisting of 3 cycles of gemcitabine. This was followed by chemoradiation that included 45 Gy in 25 fractions with concurrent capecitabine. She completed adjuvant therapy in October 2014, and as of March 2015, she has no clinical evidence of disease recurrence(Fig. 4) and a Ca19-9 of 28.5.

## Discussion

Invasive ductal carcinoma of the pancreas remains one of the most incurable malignancies with poor overall survival and high recurrence rates despite aggressive surgical and adjuvant therapies. Most of these recurrences occur within the first 2 years and are associated with distant metastatic disease, limiting the role of repeat surgery.

That said, there are rare cases of late recurrences or metachronous lesions that are resectable. There is, however, limited data in the literature to guide treatment for these patients. Much of the literature regarding repeat pancreatectomy for recurrent pancreatic cancer has been limited to case reports. Over the last 10 years, eight additional cases have been described [8–12]. Details of these other case reports are outlined in Table 1.

**Table 1:** Case reports of remnant pancreatectomy

Patient no.	Author	Age	Gender	Initial surgery	DFI (months)^a^	Second surgery	Follow-up
1	Dalla Valle et al. [9]	63	Male	PD^b^	12	DP	Alive, 24 months
2	Takamatsu et al. [8]	63	Male	PD	43	DP	Alive, 10 months
3	Miura et al. [10]	72	Female	PD	29	DP plus liver resection	Dead, 5 months
4	Miura et al. [10]	52	Female	PD	22	DP	Dead, 44 months
5	Koizumi et al. [11]	65	Male	PD	85	DP	Alive, 10 months
6	Koizumi et al. [11]	67	Male	DP^c^	25	Proximal remnant pancreatectomy	Alive, 8 months
7	Ogino et al. [12]	63	Female	PD	66	DP	Alive, 13 months
8	Ogino et al. [12]	56	Male	PD	37	DP	Alive, 7 months
9	Present case	73	Female	PD	48	DP	Alive, 11 months

^a^Disease-free interval

^b^Pancreaticoduodenectomy

^c^Distal pancreatectomy

In addition to these case reports, there have been sporadic retrospective reviews of treatment for recurrent pancreatic carcinoma.

The first of these published by Kleef et al. [13] included 30 patients with recurrent pancreatic ductal carcinoma, 15 who underwent repeat curative intent surgery, and 15 who did not have a repeat resection. The median time from initial surgery to recurrence was 12 months. Data from this study showed a median survival of 17 months in the group that was resected compared to 9.4 months in those who were not, with statistically significant survival improvement in those patients who were resected after a disease-free interval of greater than 9 months. A subsequent study by Lavu et al. [14] looked at a series of 11 patients from a single institution that underwent repeat pancreatectomy. Of these, six were for recurrent pancreatic ductal adenocarcinoma. They reported minimal complications and a median survival of 17.5 months.

A third, larger study from Germany [15] examined a series of 97 patients with pancreatic cancer recurrence. Of these, 57 had an isolated local recurrence and 41 were found to be resectable. In this study, there was a significant survival advantage in those undergoing repeat resection with median survival of 16.4 versus 9.4 months in those that were resectable or unresectable, respectively. In addition, these authors found that the ability to achieve an R0 resection was critical. The 16 patients who had an R0 resection at the second operation had a median survival of 30 months.

The most recent study evaluating the role of repeat pancreatectomy for recurrent pancreatic cancer by Miyazaki et al. [16] evaluated 170 patients from Japan with recurrent pancreatic cancer. Sixty-seven of these had isolated recurrences within the pancreatic remnant and 11 ultimately underwent re-resection. Consistent with the previous reports, they found improved median survival of 25 months with repeat resection versus 9.3 months in those not resected.

While these four studies all show similar results, with improved median survival in resectable cases of recurrent pancreatic cancer, there is some conflicting data in the literature. In particular, a study from MD Anderson Cancer Center [17] looked at the results of selective operation for locally recurrent or metastatic pancreatic cancer. This study showed little benefit in resecting local recurrences in the pancreas even after a disease-free interval on over 20 months. This study only demonstrated a benefit for metastasectomy of lung metastases in select patients with long disease-free interval.

One difficulty in evaluating patients who develop cancer in the remnant pancreas is determining if it represents local recurrence or a second primary. Previous studies have shown that the rate of multifocal carcinomas within the pancreas may as high as 32 % [18], and microscopic secondary lesions may contribute to the high rates of rapid local recurrence frequently seen with this disease. In addition, up to 16 % of patients will develop second cancers in the pancreas even after disease-free survival after surgery of 5 years [3]. While it is difficult to determine weather cancer in the remnant pancreas is a local recurrence or metachronous second primary, in the case presented here, we favor that this was a true second primary. This is largely due to a long disease-free interval with no rise in the patient’s tumor markers until the second tumor developed.

New advancements in the use of adjuvant and neoadjuvant chemotherapy and radiation have improved survival in pancreatic cancer. However, there remains some controversy regarding the sequencing of therapy for patients who develop recurrent disease within the pancreatic remnant. The NCCN has no clear guidelines regarding surgical treatment of pancreatic remnant carcinoma [19]. In the case presented here, it was felt that curative intent surgery should be attempted as the first-line therapy as there was no evidence of CT or PET-CT of metastatic disease and the lesion appeared completely resectable. In addition, the long disease-free interval suggested an indolent disease course that could potentially be cured with surgery. Postoperative chemotherapy and chemoradiation was given in the adjuvant setting and has resulted in the patient being disease free now for 11 months.

## Conclusions

In conclusion, remnant pancreatectomy remains safe and can result in prolonged disease-free survival in highly selected patients with cancer in the remnant pancreas after previous surgery for pancreatic adenocarcinoma. While the case reported here has resulted in no disease recurrence to date, the short follow-up precludes us from drawing any conclusions about cure in this patient.

## Consent

Written informed consent was obtained from the patient for publication of this case report and any accompanying images. A copy of the written consent is available for review by the Editor-in-Chief of this journal.
